# Primary lung cancer in children and adolescents

**DOI:** 10.1007/s00432-024-05750-1

**Published:** 2024-05-02

**Authors:** Qiuming Chen, Jun Cheng, Luming Wang, Xiayi Lv, Jian Hu

**Affiliations:** Department of Thoracic Surgery, The First Affiliated Hospital, Zhejiang University School of Medicine, Hangzhou, Zhejiang China

**Keywords:** Primary lung cancer, Children, Adolescents, Ground glass opacity, Solid nodule

## Abstract

**Purpose:**

Primary lung cancer is extremely rare in children and adolescents. The aim of this study is to clarify clinical features and outcomes of primary lung cancer in children and adolescents.

**Methods:**

Young patients (aged ≤ 20 years) diagnosed as primary lung cancer between 2012 and 2023 were retrospective reviewed. According to radiological appearance of the nodules, they were divided into solid nodule (SN) group and ground glass opacity (GGO) group.

**Results:**

A total of 74 patients were identified, with a median age at diagnosis of 18 years old (range: 11–20), including 7 patients in SN group and 67 patients in GGO group. In the GGO group, none of the nodules enlarged or changed during an average surveillance period of 10.8 months before surgery, except one. Wedge resection was the most common procedure (82.1%), followed by segmentectomy (16.4%) and lobectomy (1.5%). Histopathological analysis revealed that 64.2% of GGO nodules were adenocarcinoma in situ and minimally invasive adenocarcinomas, while the remaining 35.8% were invasive adenocarcinomas. Mutational analysis was performed in nine patients, with mutations identified in all cases. After a mean follow-up period of 1.73 ± 1.62 years, two patients in the SN group died due to multiple distant metastases, while all patients in the GGO group survived without recurrence. The overall survival (100%) of the GGO group was significantly higher than SN group (66.7%).

**Conclusions:**

Primary lung cancer in children and adolescents are rare and histopathological heterogeneous. Persistent GGO nodules may indicate early-stage lung adenocarcinoma in children and adolescents.

## Introduction

Primary lung cancer remains one of the most common adult cancers and is the leading cause of cancer-related mortality world-wide (Sung et al. 2021). However, the incidence of primary lung cancer is rare in young people, especially in children, which estimated at one per two million, accounting for 0.2% of all malignant tumors in this age group (Neville et al. 2009). Knowledge of pediatric lung cancer was primarily derived from individual case reports, small series of distinct histopathologic tumor subsets, a mix of benign and malignant tumors (Yu et al. 2010; Rojas et al. 2015; Voggel et al. 2021). Limited data have reported the clinical characteristics and prognosis of primary lung cancer in children and adolescents. With the coronavirus disease 2019 (COVID-19) pandemic, an increasing number of pulmonary nodules were incidentally detected in lots of people, including some children and adolescents (Wu et al. 2021). These pulmonary nodules remain a diagnostic challenge in children and adolescents, as there are numerous types of appearance and no commonly accepted features to distinguish between benignity and malignancy. Therefore, we conducted a retrospective study to clarify its clinical features and prognosis in patients under 20 years of age who underwent curative surgery for primary lung cancer.

## Patients and methods

### Study design and participants

This study conformed to the Declaration of Helsinki on Human Research Ethics standards and was approved by the institutional ethical review board of our institution (No. 2023-0488). Written patients informed consent was waived by the Institutional Review Board. Patients under 20 years old who underwent curative surgery and diagnosed with primary lung cancer at our institution between January 2012 and March 2023 were retrospectively reviewed. Clinical information was obtained from the patient’s medical record, including age, sex, smoking, family history of cancer, time from nodule detection to surgery, comorbidities, radiographical characteristics, tumor location, surgical procedures and histologic findings.

### Radiological and histologic evaluation

All the pulmonary nodules were incidentally detected by chest CT because of common respiratory symptoms such as cough, pneumonia, or shortness of breath, especially after the COVID-19 pandemic. All of them were confirmed by serial chest CT. High-resolution CT was performed at full inspiration to evaluate the entire lung, and contrast-enhanced CT scan was also performed when appropriate. Each nodule on preoperative CT scan was reviewed by experienced chest radiologists and thoracic surgeons. The diameter of pulmonary nodule was measured as the maximum diameter in the axial plane on the lung window setting even with multiple nodules. For a patient with multiple nodules, the highest T nodule was recorded. Ground glass opacity (GGO) was defined as a hazy, opaque increase in lung attenuation that did not obscure the underlying vascular markings. A solid nodule (SN) was defined as a nodule without any GGO component. According to the radiological appearance of the patient’s nodules, they were divided into SN group or GGO group. The GGO group was further subdivided into pure GGO and mixed GGO subgroups based on the presence or absence of a solid component. Three patients with SN and diameter exceeding 20 mm were preoperative diagnosed with bronchoscopic biopsy, while other patients didn’t receive preoperative biopsy.

### Surgical approach

All patients underwent video-assisted thoracoscopic surgery with combined intravenous and inhalation general anesthesia and double-lumen endotracheal intubation. The specific location of the GGO was judged by anatomical location or finger exploration. The specific surgical plan procedures were selected by surgeons after comprehensive evaluation based on the location of the nodule and the intraoperative frozen section diagnosis, including wedge resection, segmentectomy and lobectomy. Systematic lymph node dissection or sampling was performed.

### Follow-up

Postoperative surveillance was performed with physical examination, blood analysis, and chest CT every 6–12 months during the first 2 years, and annually for subsequent years. The follow-up time was calculated from the day after surgery and was followed up until April 2023. The overall survival (OS) was defined as the interval from the date of surgery to the date of death from any cause or last follow-up. Recurrence free survival (RFS) was defined as the interval from the date of surgery to the date of primary recurrence or metastasis.

### Statistical analysis

Descriptive statistics were used to present data as means, medians, or counts and percentages, as appropriate. Statistical analyses were performed by using SPSS 26.0 Software (IBM Corp, Armonk, NY, USA). Pearson’s chi-square test or Fisher’s exact test was used for comparative analyses of categorical variables, when appropriate. Mann Whitney U were used to compare inter-group differences in continuous variables. Kaplan–Meier curves were used to estimate OS. A value of P < 0.05 was considered statistically significant.

## Results

### Patient characteristics

**Fig. 1:**
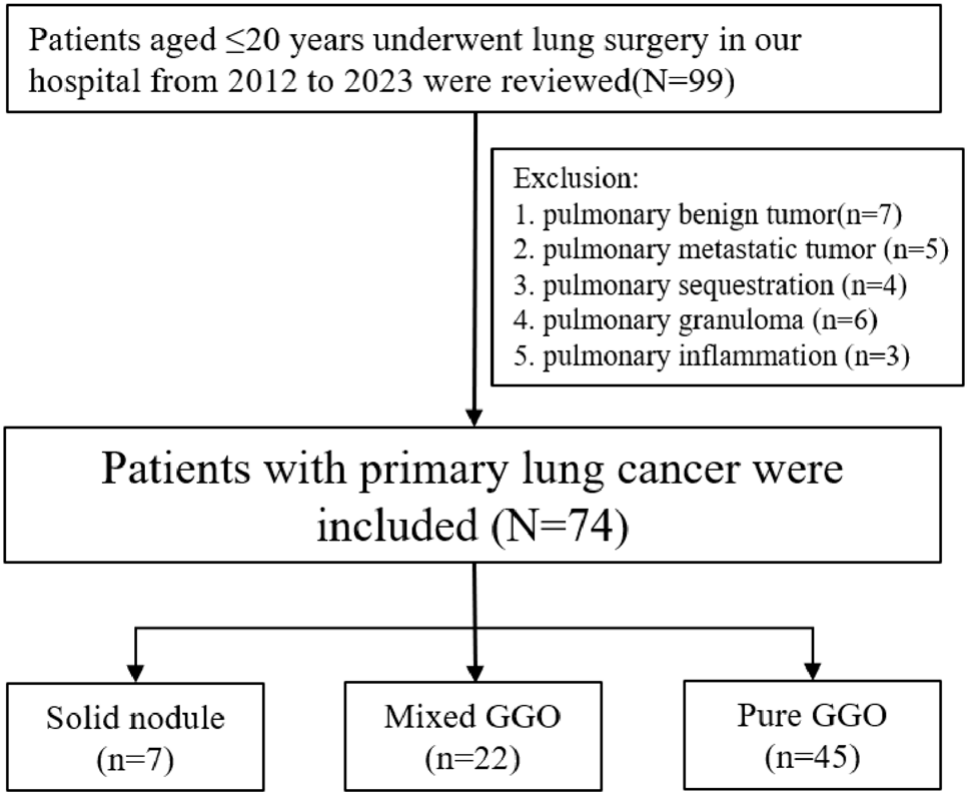
Flow chart for patient selection in this study

**Fig. 2:**
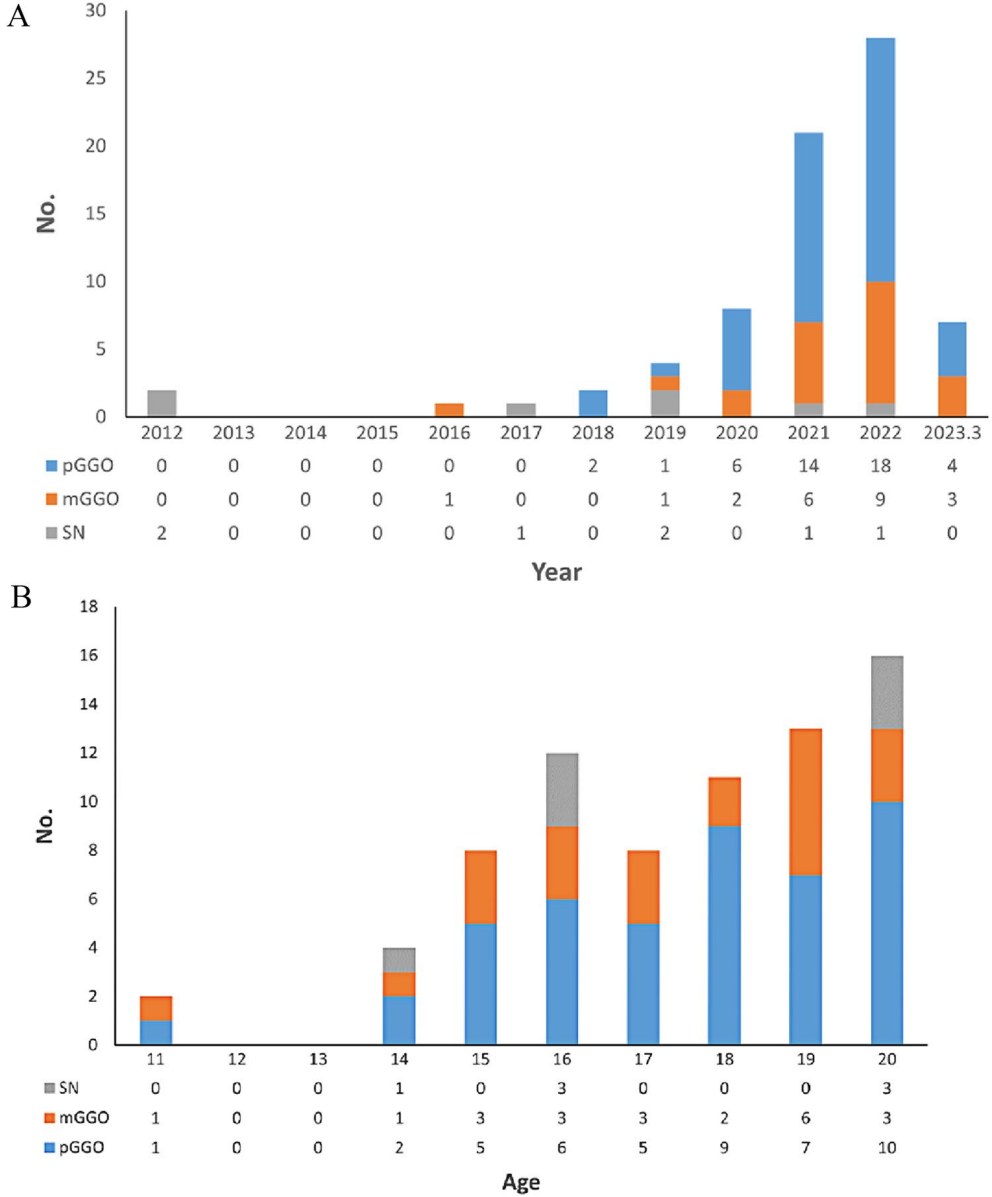
**A** The time distribution of the number of patients enrolled in the study. **B** The age distribution of the number of patients enrolled in the study

A total of 74 patients were identified with a median age at diagnosis of 18 years old (range 11–20 years old), including 44 females and 30 males. The inclusion and exclusion process were shown in Fig. 1. Figure 2 shows the time and age distribution of patient enrollment in the study, including 7 patients in SN group and 67 patients in GGO group (45 for pGGO group and 22 for mGGO group). There are 2 patients aged 11, 4 patients aged 14, 8 patients aged 15, and 12 patients aged 16. None of the patients had a history of smoking. Only one patient in the pGGO group had a family history of lung adenocarcinoma. Clinical features comparison between patients with GGO group and SN group were showed in Table 1, which showed SN group had larger tumor size and more lobectomy resection.

**Table 1:** Clinical features comparison between patients with ground glass opacity nodules and solid nodules

	GGO (N = 67)	SN (N = 7)	P
Age (years ± SD)	17.45 ± 2.16	17.43 ± 2.51	0.982
Male	27 (40.3%)	3 (42.9%)	1.000
Current or former smoking	0	0	
Family history of cancer	1 (1.5%)	0	1.000
Tumor size (mean ± SD, mm)	8.52 ± 1.96	22.83 ± 13.67	**0.000**
Nodule location			0.325
Right upper lobe	12 (17.9%)	3 (42.9%)	
Right middle lobe	10 (14.9%)	0	
Right lower lobe	17 (25.4%)	2 (28.6%)	
Left upper lobe	14 (20.9%)	0	
Left lower lobe	14 (20.9%)	2 (28.6%)	
Surveillance time (months)	10.80 ± 12.17	2.31 ± 4.52	**0.008**
Operative time (min)	66.67 ± 35.48	118.71 ± 35.48	**0.001**
Operative procedure			**0.000**
Lobectomy	1 (1.5%)	6 (85.7%)	
Segmentectomy	11 (16.4%)	0	
Wedge resection	55 (82.1%)	1 (14.3%)	
Postoperative hospital stays (day)	3.8 ± 1.49	4.00 ± 1.00	0.822

*GGO* ground glass opacity, *SN* solid nodule

**Fig. 3:**
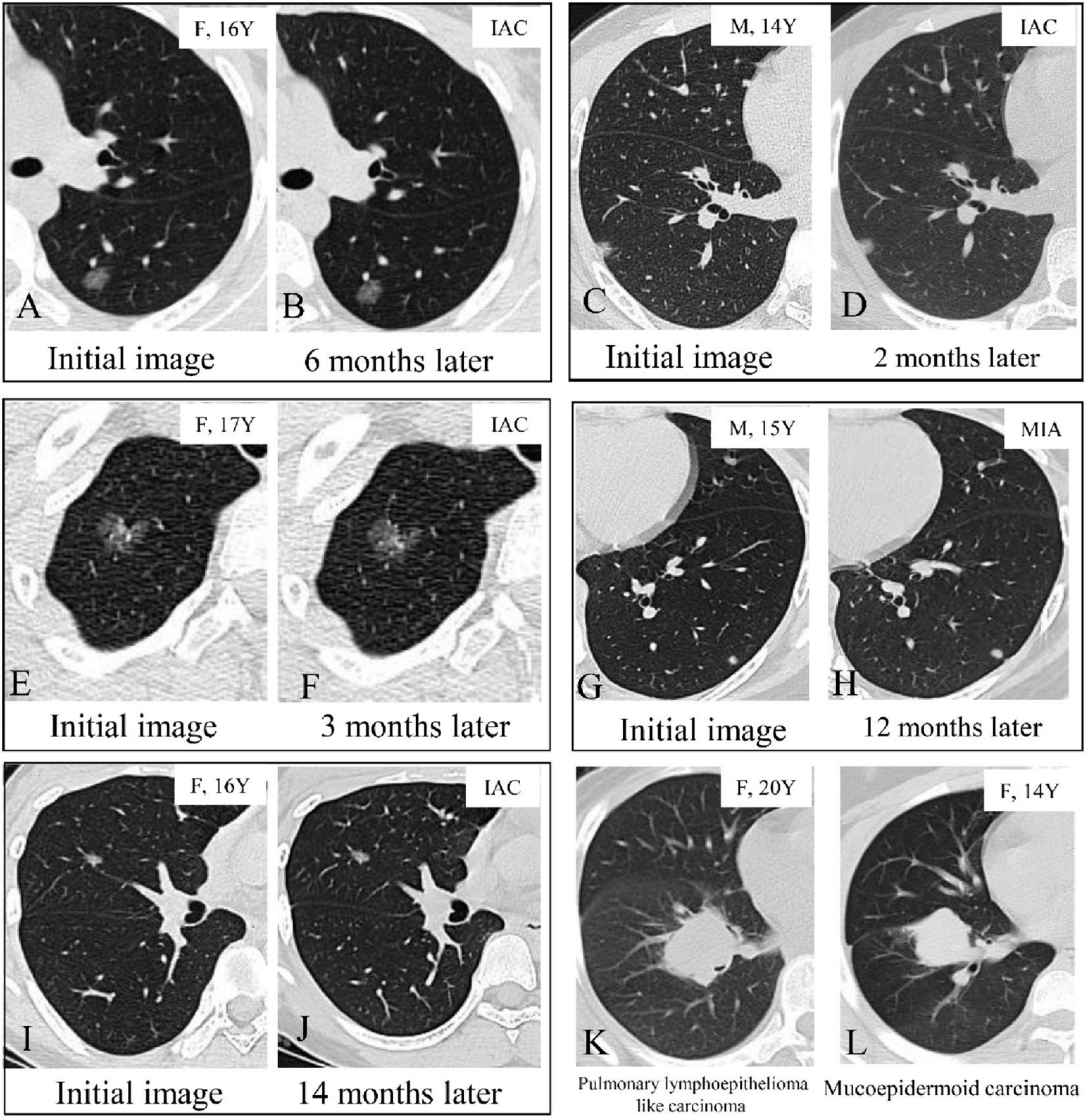
Examples of pulmonary nodules. Examples of pure ground glass opacity nodules that remained unchanged after 7 months and pathological diagnosis was invasive adenocarcinoma (IAC) (**A**, **B**). Examples of mixed ground glass opacity nodules that remained unchanged after 2 months (**C**, **D**, invasive adenocarcinoma) and 3 months (**E**, **F**, invasive adenocarcinoma) of follow-up. Examples of solid nodule that remained unchanged after 12 months and pathological diagnosis was minimally invasive adenocarcinoma (MIA) (**G**, **H**). **I**, **J**, A ground glass opacity nodule that enlarged from 0.6 cm (**I**) to 0.9 cm (**J**) after 14 months of follow-up. The final pathology was acinar predominant adenocarcinoma, pT1aN0M0. **K** A 20- year-old woman with a solid nodule was diagnosed pulmonary lymphoepithelioma like carcinoma, pT2aN2M0 and died due to multiple distant metastases 1.5 years after surgery. **L** A 14- year-old woman with a solid nodule is diagnosed mucoepidermoid carcinoma, pT2aN2M0 and stays alive

The clinical characteristics of the included patients in the GGO group are summarized in Table 2. The median observation period before surgery was 6 months (ranging from 1 to 60 months). During the surveillance period, only one lung nodule in the mGGO group enlarged in the diameter from 6 to 9 mm after 1 year of surveillance (Fig. 3). The average tumor diameter of SN nodules was 22.83 ± 13.67 mm, followed by mGGO nodules (13.16 ± 5.84 mm). The diameter of SN nodules was more distributed in the range of >10 mm, with nodules ≥15 mm accounting for 71.4%, while the diameter of GGO nodule was mostly ≤10 mm, accounting for 86.6%. Radiologically, the majority of GGO nodules manifested as pure GGO (73.8%) or mixed GGO (26.2%). The most common location of GGO was the right lower lobe (17, 25.4%), followed by the left lower lobe and the left upper lobe (14, 20.9%). The majority of GGO nodules were located peripherally (93.9%), and subpleural nodules were present in 38 (56.7%) patients.

**Table 2:** Clinical features of patients with ground glass opacity nodules

	N = 67	pGGO (N = 45)	mGGO (n = 22)	P
Age (years ± SD)	17.45 ± 2.16	17.56 ± 2.10	17.23 ± 2.31	0.562
Age ≤ 18	41 (61.2%)	28 (62.2%)	13 (59.1%)	
18 < Ag ≤ 20	26 (38.8%)	17 (37.8%)	9 (40.9%)	
Male	27 (40.3%)	18 (40.0%)	9 (40.9%)	0.943
Current or former smoking	0	0	0	
Family history of cancer	1 (1.5%)	1 (2.2%)	0	1.000
Subpleural nodules	38 (56.7%)	22 (48.9%)	16 (72.7%)	0.064
Tumor size (mean ± SD, mm)	8.52 ± 1.96	8.33 ± 1.74	8.9 ± 2.35	0.263
≤ 10	58 (86.6%)	39 (67.2%)	19 (32.8%)	
> 10	9 (13.4%)	6 (66.7%)	3 (33.3%)	
Nodule location				0.758
Right upper lobe	12 (17.9%)	7 (15.6%)	5 (22.7%)	
Right middle lobe	10 (14.9%)	7 (15.6%)	3 (13.6%)	
Right lower lobe	17 (25.4%)	13 (28.9%)	4 (18.2%)	
Left upper lobe	14 (20.9%)	8 (17.8%)	6 (27.3%)	
Left lower lobe	14 (20.9%)	10 (22.2%)	4 (18.2%)	
Surveillance time (months)	10.80 ± 12.17	10.32 ± 11.26	11.77 ± 14.04	0.675
≤ 1 month	4 (6.0%)	3 (6.6%)	1 (4.5%)	
> 1 month, ≤ 6 months	33 (49.3%)	21 (46.7%)	12 (54.5%)	
> 6 months, ≤ 1 year	15 (22.4%)	12 (26.7%)	3 (13.6%)	
>1 year	15 (22.4%)	9 (20.0%)	6 (27.2%)	
Operative time (min)	66.67 ± 35.48	68.02 ± 37.58	63.95 ± 31.51	0.664
Operative procedure				**0.049**
Lobectomy	1 (1.5%)	0	1 (4.5%)	
Segmentectomy	11 (16.4%)	10 (22.2%)	1 (4.5%)	
Wedge resection	55 (82.1%)	35 (77.8%)	20 (90.9%)	
Postoperative hospital stays (day)	3.8 ± 1.49	4.00 ± 1.66	3.41 ± 1.01	0.130
Histological type				**0.001**
AIS	6 (9.0%)	6 (13.3%)	0	
MIA	37 (55.2%)	29 (64.4%)	8 (36.4%)	
IAC	24 (35.8%)	10 (22.2%)	14 (63.6%)	
Predominant subtype				0.429
Lepidic	17 (25.3%)	6 (13.3%)	11 (50.0%)	
Acinar	6 (9.0%)	3 (6.7%)	3 (13.6%)	
Papillary	1 (1.5%)	1 (2.2%)	0	
Pathological N1/N2	0	0	0	

*AIS* adenocarcinoma in situ, *mGGO* mixed ground glass opacity, *MIA* minimally invasive adenocarcinoma, *IAC* invasive adenocarcinoma, *pGG*O pure ground glass opacity

### Surgery results

All the patients in the SN group underwent lobectomy except two patients who underwent wedge resection. The median postoperative hospital stay was 4 days (ranging from 2 to 6 days). All of them had a smooth postoperative recovery. All patients in the GGO group underwent video-assisted thoracoscopic surgery without preoperative hook-wire localization. A total of 72 GGO nodules were successfully located and resected, including two pulmonary nodules resected respectively in five patients. Wedge resection was the most frequent procedure in 55 (82.1%) patients, while segmentectomy was performed in 11 (16.4%) patients, including the superior segment of the lower lobe in 9 patients, inner segment of the right middle lobe in one patient, and left upper lobe posterior segment in one patient. Only one patient with 9 mm mGGO nodule underwent a right middle lobectomy and the pathology examination was moderately differentiated with infiltrating adenocarcinoma. Furthermore, she had undergone liver sarcoma resection 1 year ago, thyroid cancer surgery 6 months ago, and brain schwannoma resection surgery 1 month ago. The mean operative time was 66.67 ± 35.48 min (median: 58; range: 29–195 min). The median postoperative hospital stay was 4 days (range: 2–12 days). Only one patient with pGGO after wedge resection had prolonged air leak and stayed in the hospital for 12 days. Compared to patients receiving segmentectomy or lobectomy, patients receiving wedge resection experienced significantly shorter surgery duration, fewer chest tube dwelling days, less chest tube output, and shorter postoperative hospital stay (Table 3).

**Table 3:** Comparison between wedge resection and segmentectomy or lobectomy for patients with ground glass opacity nodules

	Wedge resection (N = 55)	Segmentectomy or lobectomy (N = 12)	P
Age (year ± SD)	17.33 ± 2.25	18.00 ± 1.70	0.331
Male	25 (45.5%)	2 (16.7%)	0.104
Subpleural nodules	38 (69.1%)	0	**0.000**
Surveillance time (months)	10.52 ± 10.72	12.08 ± 17.85	0.690
Tumor diameter (mm)	8.56 ± 1.93	8.33 ± 2.19	0.775
Operative time (min)	57.00 ± 23.42	110.17 ± 47.67	**0.000**
Pure ground glass opacity	35 (63.6%)	10 (83.3%)	0.310
Postoperative chest tube duration (day)	2.48 ± 1.42	3.00 ± 1.21	0.246
Postoperative hospital stays	3.72 ± 1.52	4.17 ± 1.34	0.354
Nodule location			0.504
Right upper lobe	11 (20.0%)	1 (8.3%)	
Right middle lobe	8 (14.5%)	2 (16.7%)	
Right lower lobe	13 (23.6%)	4 (33.3%)	
Left upper lobe	13 (23.6%)	1 (8.3%)	
Left lower lobe	10 (18.2%)	4 (33.3%)	
Histological type			0.432
AIS	6 (10.9%)	0	
MIA	29 (52.7%)	8 (66.7%)	
IAC	20 (36.4%)	4 (33.3%)	

*AIS* adenocarcinoma in situ, *MIA* minimally invasive adenocarcinoma, *IAC* invasive adenocarcinoma

### Pathological characteristics and mutational analysis

The SN group showed different histopathologic tumor types compared to the GGO group, including mucoepidermoid carcinoma (n = 2), pneumonia myofibroblastoma (n = 2), primary pulmonary rhabdomyosarcoma (n = 1), pulmonary lymphoepithelioma-like carcinoma (n = 1), and minimally invasive adenocarcinoma (MIA, n = 1), which is shown in (Table 4). Only one patient with pulmonary lymphoepithelioma carcinoma-like had mediastinal lymph node metastasis. The SN group displayed significantly worse pathological subtypes compared with the GGO group.

**Table 4:** Clinical features of patients with solid nodule

Patient	Age	Sex		Histotypes	Tumor size (mm)	Surgical procedure	TNM	Lob	POD	Status at last FU	Follow up time (years)
1	20		F	Pneumonia myofibroblastoma	18	Lobectomy	T1bN0M0	RUL	5	Alive	3.4
2	20	F		Pneumonia myofibroblastoma	10	Lobectomy	T1aN0M0	RUL	4	Alive	2.2
3	20	F		Pulmonary lymphoepithelioma-like carcinoma	30	Lobectomy	T2aN2M0	RLL	6	Metastasis and dead	1.5
4	14	M		Mucoepidermoid carcinoma	37	Lobectomy	T2aN0M0	RLL	5	Alive	3.4
5	16	M		Primary pulmonary rhabdomyosarcoma	17	Wedge	T1bN0M0	RUL	6	Alive	11
6	16	M		Mucoepidermoid carcinoma	37	Lobectomy	T2aN0M0	LLL	5	Metastasis and dead	1
7	16	F		Minimally invasive adenocarcinoma	6	Wedge	TmiN0M0	LLL	3	Alive	0.7

*FU* follow up, *LRL* left upper lobe, *LLL* left lower lobe, *RUL* right upper lobe, *RML* right middle lobe, *RLL* right lower lobe, *POD* postoperative days

The pathological analysis results revealed that 64.2% of GGO group were adenocarcinoma in situ (AIS) and MIA, while the remaining 35.8% (24/67) of the nodules were invasive adenocarcinoma (IAC), consisting of 17 (70.8%) patients with lepidic pattern-predominant adenocarcinomas, 6 (25%) patients with acinar pattern-predominant adenocarcinomas and only 1 (4.3%) patient with papillary pattern-predominant adenocarcinomas. None of the patients had adenocarcinomas dominant in the micropapillary pattern. IAC was more prevalent in patients with mGGO (P = 0.001). No metastasis was found in any of the lymph nodes examined.

Genetic mutations were detected in all 9 patients who underwent genetic testing in GGO group, including ERBB2 mutations in 4 patients, EGFR mutations in 3 patients, ALK mutation in 1 patient and RET mutation in 1 patient. EGFR mutations included EGFR 19 Del mutations in two patients with IAC and EGFR exon20 insertion mutation in a female patient with MIA.

### Follow-up and survival

**Fig. 4:**
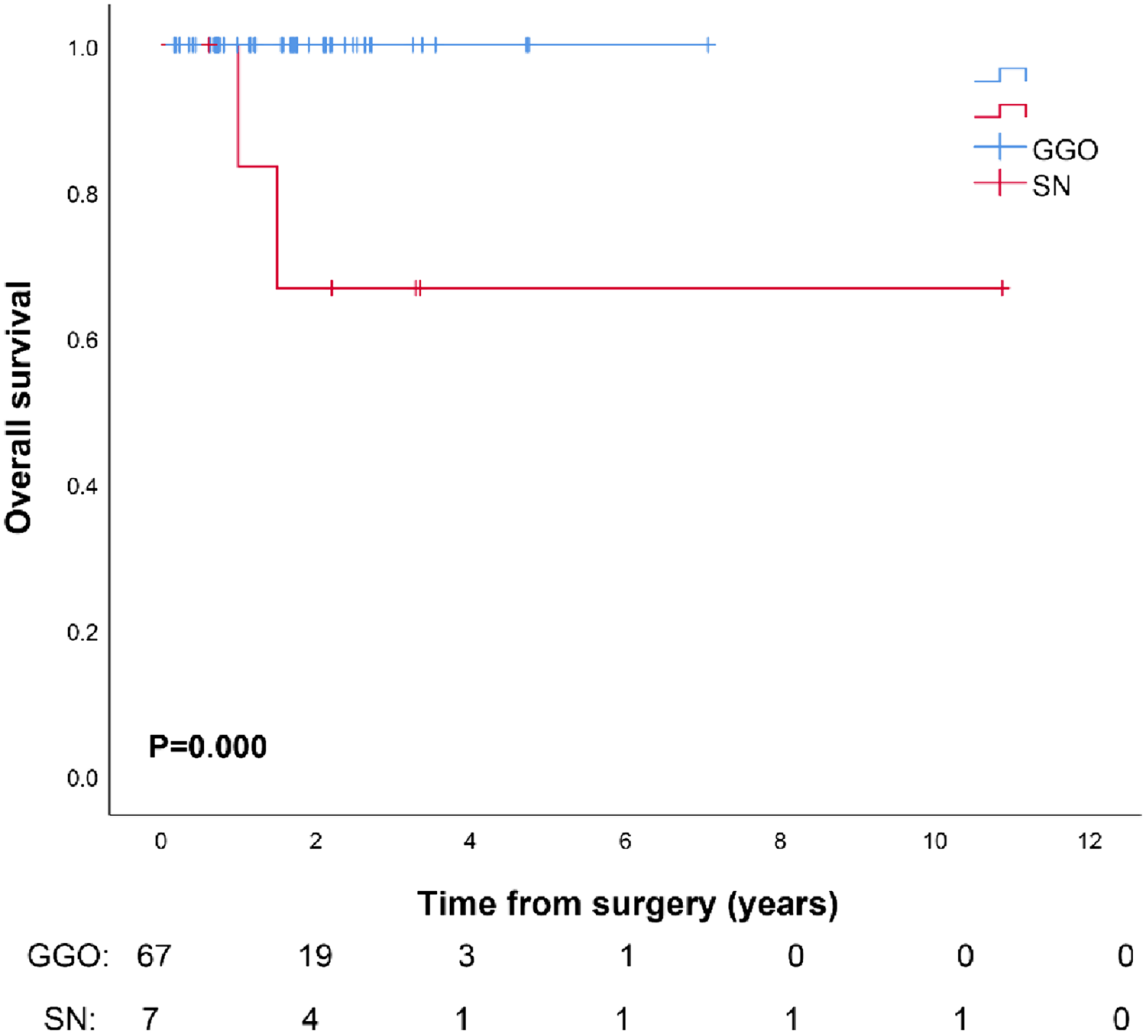
Overall survival rates of patients between GGO group and SN group. *OS* overall survival, *GGO*: ground glass opacity, *SN* solid nodule

Until April 2023, all 74 patients were successfully followed, with an average follow-up time of 1.73 ± 1.62 years (range: 0.16–10.87 years). Two patients in the SN group died from multiple distant metastases, one patient with pulmonary lymphoepithelioma-like carcinoma died 1.5 years after surgery, and the other patient with mucoepidermoid carcinoma died 1 year after surgery. All patients of GGO group and the other five patients of SN group survived without recurrence. The results of the survival analysis showed that the 3-year OS and RFS of the GGO group were 100%, while the 3-year OS and RFS of the SN group were 66.7 ± 19.2% and 66.7 ± 19.2%, respectively (Fig. 4).

## Discussion

Primary lung cancer was quite rare in children and adolescence. Diagnose and treatment of primary lung cancer in children and adolescents can be challenging due to its rarity and non-specific symptoms at presentation. Many pulmonary nodules were found incidentally on radiologic studies requested for completely unrelated medical issues (Liang and Lee 2022). Currently, the literature primarily consists of case series about primary lung cancer in children and adolescents (Youlden et al. 2020). Michael et al. reported on 38 patients under 18 years old with primary lung cancer, highlighting the rarity of this disease (Abele et al. 2022). Previous studies (Neville et al. 2009; Rojas et al. 2015) showed that primary lung cancer had a poor prognosis in children, with 26% of the 5-year survival rate, due to nonspecific manifestations that frequently result in a delayed diagnosis and higher prevalence of metastatic disease. Similar to previous studies, our study identified seven patients with solid nodules diagnosed as primary lung cancer in past decade, and 28.5% of them died from multiple distant metastases. It indicated that tumor histology was the most reliable predictor of survival in pediatric patients with primary lung cancer.

As a non-high-risk population for lung cancer, there is no regular health examination of LDCT for children and adolescents. However, more and more pulmonary nodules are being accidentally detected in young people who come to the hospital for common respiratory symptoms such as cough, pneumonia, or shortness of breath, especially after the COVID-19 pandemic (Renne et al. 2015; Samim et al. 2017). Zhang et al. found many GGO-featured lung adenocarcinomas have been detected in China in lung cancer screening, which tend to occur in young, female, nonsmoking populations (Zhang et al. 2020a). Lung adenocarcinoma is extremely rare in the pediatric population, comprising only 8% of lung cancer in childhood while it is the most common malignant lung cancer in adults (Rojas et al. 2015). A systematic review of pediatric lung adenocarcinoma included 48 studies comprising 62 patients with adenocarcinoma and 21 cases of AIS (Balzer et al. 2018). Similar with Wu et al. study (Wu et al. 2021) which reported 12 patients diagnosed as GGO featured lung cancer, 67 patients with GGO nodules were diagnosed as preinvasive or invasive adenocarcinoma and clinical stage was early in this study. These findings indicate that more attention should be paid to persistent GGO accidentally detected in children and adolescents.

An important question raised by these findings is why lung cancer occurs in young patients. Tobacco is the leading risk factor for lung cancer in adult patients. However, there was no smoker in our study while only one patient was reported to be a smoker among 12 teenagers in Wu’s study (Wu et al. 2021). Long-term exposures to various environmental factors, such as second-hand smoke, air pollution, kitchen fumes, or asbestos, have been considered as potential elevated risk factors for lung cancer (Hill et al. 2023). However, exposure to those risk factors both in terms of time and amount is probably insufficient to cause lung cancer in these children and adolescents. Furthermore, only one patient had a family history of lung cancer, while none of the 12 teenagers with lung adenocarcinoma had a family history of lung cancer in Wu’s study (Wu et al. 2021), which could be related with small sample size. But it also serves as a reminder that young patients with a family history of cancer may be at increased risk for developing lung cancer. No specific gene mutations have been identified in pediatric lung cancer, although the prevalence of some types of mutations differs with age (Chen et al. 2019). Cai et al. found young lung cancer patients are typically highly actionable, with approximately 88% of patients harboring at least one targetable genetic alteration (Cai et al. 2021). This study also showed that all patients who underwent molecular mutation testing had mutation genes, indicating that these gene mutations play an important role in the malignant progression to lung cancer in children and adolescents. More research is urgently needed to identify risk factors and explore the pathogenesis of lung cancer that developed in such a young age.

After incidentally detected by CT, the first issue of pulmonary nodules in children and adolescents is diagnosis and treatment. Overdiagnosis and overtreatment are major concerns because GGO is gradually considered an indolent disease. A period of follow-up is the optimal strategy to reduce overdiagnosis and overtreatment (Silva et al. 2018). But the appropriate duration of follow-up is unclear because there is still no reliable way to forecast its development. There are no widely accepted standards for assessing pulmonary nodules in children and adolescents currently (Liang and Lee 2022), so treatment guidelines are extrapolated from adult guidelines (Cardillo and Petersen 2023). According to the Fleischner Society Guidelines for Pulmonary Nodules (Macmahon et al. 2017), patients with persistent GGO < 6 mm should undergo a follow-up CT scan every 1–2 years until 5 years or consider surgical resection if the solid component or growth develops. Lee et al. reported that 13.0% of GGO growth was identified during a follow-up of 136 months, even when smaller than 6 mm and stable for 5 years (Lee et al. 2019). This indicates that some GGOs still need to be monitored after 5 years, so the radiation exposure from frequent follow-up CT scans should not be ignored, which may be harmful to children and adolescents (Bach et al. 2012). Considering that the majority of these patients are GGO featured early-stage lung cancer, the interval of follow-up can be extended more than routine in children and adolescent. Understanding the natural history of GGO can assist in determining optimal treatment timing and management of these nodules.

It remains controversial whether persistent GGO in children and adolescents should be surgically resected or followed-up. Frequent follow-up CT scans may expose patients to a large dose of radiation and cause anxiety for certain patients and families. Surgery may result in complications and pulmonary functional loss because of lung resection. In fact, with the progress of minimally invasive technology, the overwhelming majority of patients undergoing sublobar resection of GGO recover quickly and return to normal social lives within 2–4 weeks after surgery (Zhang et al. 2020a; Li et al. 2023). Furthermore, GGO-lung cancer has an excellent prognosis after resection, especially for patients with AIS or MIA, which showed that 10-year OS is close to 100% (Yotsukura et al. 2021). If the GGO progress in size or solid component during follow-up, the extent of surgical resection may need to be increased and the patient’s prognosis might deteriorate. Additionally, our study showed that 35.8% of the GGO nodules were invasive adenocarcinoma. Due to the anxiety and radiation exposure associated with long-term follow up, some patients with persistent GGO would prefer surgery rather than a wait-and-see strategy. These indicates that the treatment of pulmonary nodules in children and adolescents requires individual treatment strategies after thoughtful discussion among the patient, parents and doctors. Patient age and nodule location should be taken into account when deciding on aggressive surgical intervention for persistent GGO (Sim et al. 2014; Zhang et al. 2020b). Early intervention is advised for young individuals with peripheral GGO nodules that can be entirely resected by sublobar resection. Conversely, central GGO nodules that necessitate lobectomy should be followed up. In this study, we also found that patients with persistent GGO in children and adolescents could be cured by surgery and no patient developed recurrence, supporting a less-intensive postoperative follow-up strategy.

However, there are several limitations to this study. First, this is a single-center retrospective study with intrinsic biases and selection bias cannot be avoided. Patients with similar pulmonary nodules who did not undergo surgery were not included and analyzed. The numbers of CT scans and follow-up intervals for each GGO were not constant. Second, the sample size is a very small, which prevents the adjustment of comparisons for possible confounders. Third, the follow-up period of this study was still relatively short and longer follow-up is necessary to provide a more comprehensive prognosis for patients with lung cancer in children and adolescents. Finally, the characteristics of our population may differ from those in other countries, limiting the generalizability of our findings.

## Conclusion

In conclusion, primary lung cancer in children and adolescents is rare and histopathological diverse, midterm survival is favorable but histologically dependent. Persistent GGO nodules may indicate early-stage lung adenocarcinoma in children and adolescents.

## Data Availability

The data underlying this article will be shared on reasonable request to the corresponding author.

## References

[CR1] Abele M, Bajciova V, Wright F, Behjati S, Voggel S, Schneider DT et al (2022) Primary lung carcinoma in children and adolescents: an analysis of the European Cooperative Study Group on paediatric rare tumours (expert). Eur J Cancer 175:19–30. 10.1016/j.ejca.2022.08.007 36087394 10.1016/j.ejca.2022.08.007

[CR2] Bach PB, Mirkin JN, Oliver TK, Azzoli CG, Berry DA, Brawley OW et al (2012) Benefits and harms of CT screening for lung cancer: a systematic review. JAMA 307(22):2418–2429. 10.1001/jama.2012.5521 22610500 10.1001/jama.2012.5521 PMC3709596

[CR3] Balzer B, Loo C, Lewis CR, Trahair TN, Anazodo AC (2018) Adenocarcinoma of the lung in childhood and adolescence: a systematic review. J Thorac Oncol 13(12):1832–1841. 10.1016/j.jtho.2018.08.2020 30194036 10.1016/j.jtho.2018.08.2020

[CR4] Cai L, Chen Y, Tong X, Wu X, Bao H, Shao Y et al (2021) The genomic landscape of young and old lung cancer patients highlights age-dependent mutation frequencies and clinical actionability in young patients. Int J Cancer. 10.1002/ijc.33583 33811322 10.1002/ijc.33583

[CR5] Cardillo G, Petersen RH (2023) European guidelines on the surgical management of pure ground glass opacities and part solid nodules: task force of the EACTS and ESTS. Eur J Cardiothorac Surg. 10.1093/ejcts/ezad222 37243746 10.1093/ejcts/ezad222

[CR6] Chen Z, Teng X, Zhang J, Huang K, Shen Q, Cao H et al (2019) Molecular features of lung adenocarcinoma in young patients. BMC Cancer 19(1):777. 10.1186/s12885-019-5978-5 31387567 10.1186/s12885-019-5978-5 PMC6685166

[CR7] Hill W, Lim EL, Weeden CE, Lee C, Augustine M, Chen K et al (2023) Lung adenocarcinoma promotion by air pollutants. Nature 616(7955):159–167. 10.1038/s41586-023-05874-3 37020004 10.1038/s41586-023-05874-3 PMC7614604

[CR8] Lee HW, Jin KN, Lee JK, Kim DK, Chung HS, Heo EY et al (2019) Long-term follow-up of ground-glass nodules after 5 years of stability. J Thorac Oncol 14(8):1370–1377. 10.1016/j.jtho.2019.05.005 31085340 10.1016/j.jtho.2019.05.005

[CR9] Li D, Deng C, Wang S, Li Y, Zhang Y, Chen H (2023) Ten-year follow-up results of pure ground-glass opacity-featured lung adenocarcinomas after surgery. Ann Thorac Surg. 10.1016/j.athoracsur.2023.01.014 36646243 10.1016/j.athoracsur.2023.01.014

[CR10] Liang TI, Lee EY (2022) Pediatric pulmonary nodules: imaging guidelines and recommendations. Radiol Clin North Am 60(1):55–67. 10.1016/j.rcl.2021.08.004 34836566 10.1016/j.rcl.2021.08.004

[CR11] Macmahon H, Naidich DP, Goo JM, Lee KS, Leung A, Mayo JR et al (2017) Guidelines for management of incidental pulmonary nodules detected on CT images: from the Fleischner Society 2017. Radiology 284(1):228–243. 10.1148/radiol.2017161659 28240562 10.1148/radiol.2017161659

[CR12] Neville HL, Hogan AR, Zhuge Y, Perez EA, Cheung MC, Koniaris LG et al (2009) Incidence and outcomes of malignant pediatric lung neoplasms. J Surg Res 156(2):224–230. 10.1016/j.jss.2009.03.100 19631347 10.1016/j.jss.2009.03.100

[CR13] Renne J, Linderkamp C, Wacker F, Berthold LD, Weidemann J (2015) Prevalence and configuration of pulmonary nodules on multi-row CT in children without malignant diseases. Eur Radiol 25(9):2651–2656. 10.1007/s00330-015-3675-6 25735514 10.1007/s00330-015-3675-6

[CR14] Rojas Y, Shi YX, Zhang W, Beierle EA, Doski JJ, Goldfarb M et al (2015) Primary malignant pulmonary tumors in children: a review of the national cancer data base. J Pediatr Surg 50(6):1004–1008. 10.1016/j.jpedsurg.2015.03.032 25812444 10.1016/j.jpedsurg.2015.03.032

[CR15] Samim A, Littooij AS, van den Heuvel-Eibrink MM, Wessels FJ, Nievelstein R, de Jong PA (2017) Frequency and characteristics of pulmonary nodules in children at computed tomography. Pediatr Radiol 47(13):1751–1758. 10.1007/s00247-017-3946-2 28871322 10.1007/s00247-017-3946-2 PMC5693979

[CR16] Silva M, Prokop M, Jacobs C, Capretti G, Sverzellati N, Ciompi F et al (2018) Long-term active surveillance of screening detected subsolid nodules is a safe strategy to reduce overtreatment. J Thorac Oncol 13(10):1454–1463. 10.1016/j.jtho.2018.06.013 30026071 10.1016/j.jtho.2018.06.013

[CR17] Sim HJ, Choi SH, Chae EJ, Kim HR, Kim YH, Kim DK et al (2014) Surgical management of pulmonary adenocarcinoma presenting as a pure ground-glass nodule. Eur J Cardiothorac Surg 46(4):632–636. 10.1093/ejcts/ezu007 24566849 10.1093/ejcts/ezu007

[CR18] Sung H, Ferlay J, Siegel RL, Laversanne M, Soerjomataram I, Jemal A et al (2021) Global cancer statistics 2020: globocan estimates of incidence and mortality worldwide for 36 cancers in 185 countries. Ca Cancer J Clin 71(3):209–249. 10.3322/caac.21660 33538338 10.3322/caac.21660

[CR19] Voggel S, Abele M, Seitz C, Agaimy A, Vokuhl C, Dirksen U et al (2021) Primary lung carcinoma in children and adolescents—clinical characteristics and outcome of 12 cases from the German registry for rare paediatric tumours (step). Lung Cancer 160:66–72. 10.1016/j.lungcan.2021.08.004 34418863 10.1016/j.lungcan.2021.08.004

[CR20] Wu H, Zhang Y, Hu H, Li Y, Shen X, Liu Q et al (2021) Ground glass opacity featured lung adenocarcinoma in teenagers. J Cancer Res Clin Oncol 147(12):3719–3724. 10.1007/s00432-021-03611-9 33829316 10.1007/s00432-021-03611-9 PMC8026089

[CR21] Yotsukura M, Asamura H, Motoi N, Kashima J, Yoshida Y, Nakagawa K et al (2021) Long-term prognosis of patients with resected adenocarcinoma in situ and minimally invasive adenocarcinoma of the lung. J Thorac Oncol 16(8):1312–1320. 10.1016/j.jtho.2021.04.007 33915249 10.1016/j.jtho.2021.04.007

[CR22] Youlden DR, Foresto SA, Aitken JF (2020) Primary malignant lung tumors in children: a report from the Australian childhood cancer registry, 1983–2015. Pediatr Pulmonol 55(3):719–722. 10.1002/ppul.24636 31909892 10.1002/ppul.24636

[CR23] Yu DC, Grabowski MJ, Kozakewich HP, Perez-Atayde AR, Voss SD, Shamberger RC et al (2010) Primary lung tumors in children and adolescents: a 90-year experience. J Pediatr Surg 45(6):1090–1095. 10.1016/j.jpedsurg.2010.02.070 20620301 10.1016/j.jpedsurg.2010.02.070

[CR24] Zhang Y, Fu F, Chen H (2020b) Management of ground-glass opacities in the lung cancer spectrum. Ann Thorac Surg 110(6):1796–1804. 10.1016/j.athoracsur.2020.04.094 32525031 10.1016/j.athoracsur.2020.04.094

[CR25] Zhang Y, Jheon S, Li H, Zhang H, Xie Y, Qian B et al (2020a) Results of low-dose computed tomography as a regular health examination among Chinese hospital employees. J Thorac Cardiovasc Surg 160(3):824–831. 10.1016/j.jtcvs.2019.10.145 31987625 10.1016/j.jtcvs.2019.10.145

